# Superinfections caused by carbapenem-resistant Enterobacterales in hospitalized patients with COVID-19: a multicentre observational study from Italy (CREVID Study)

**DOI:** 10.1093/jacamr/dlac064

**Published:** 2022-06-16

**Authors:** Marco Falcone, Lorenzo Roberto Suardi, Giusy Tiseo, Valentina Galfo, Sara Occhineri, Stefano Verdenelli, Giancarlo Ceccarelli, Melita Poli, Marco Merli, Davide Bavaro, Anna Carretta, Giuseppe Nunnari, Emmanuele Venanzi Rullo, Enrico Maria Trecarichi, Chiara Papalini, Antonina Franco, Rosa Fontana Del Vecchio, Vincenzo Bianco, Rodolfo Punzi, Daniela Francisci, Raffaella Rubino, Carlo Torti, Massimo Puoti, Sergio Carbonara, Antonio Cascio, Annalisa Saracino, Teresa Santantonio, Mario Venditti, Francesco Menichetti

**Affiliations:** Infectious Diseases Unit, Department of Clinical and Experimental Medicine, Azienda Ospedaliera Universitaria Pisana, University of Pisa, Pisa, Italy; Infectious Diseases Unit, Department of Clinical and Experimental Medicine, Azienda Ospedaliera Universitaria Pisana, University of Pisa, Pisa, Italy; Infectious Diseases Unit, Department of Clinical and Experimental Medicine, Azienda Ospedaliera Universitaria Pisana, University of Pisa, Pisa, Italy; Infectious Diseases Unit, Department of Clinical and Experimental Medicine, Azienda Ospedaliera Universitaria Pisana, University of Pisa, Pisa, Italy; Infectious Diseases Unit, Department of Clinical and Experimental Medicine, Azienda Ospedaliera Universitaria Pisana, University of Pisa, Pisa, Italy; Infectious Diseases Unit, Department of Clinical and Experimental Medicine, Azienda Ospedaliera Universitaria Pisana, University of Pisa, Pisa, Italy; Department of Public Health and Infectious Diseases, Sapienza University of Rome, Rome, Italy; Vittorio Emanuele II Hospital, Bisceglie, Italy; Divisione di Malattie Infettive, ASST Grande Ospedale Metropolitano Niguarda, Milan, Italy; University of Bari, Clinic of Infectious Diseases, Bari, Italy; Department of Infectious Diseases, University Hospital ‘Ospedali Riuniti’ of Foggia, Foggia, Italy; Unit of Infectious Diseases, Department of Clinical and Experimental Medicine, University of Messina, Messina, Italy; Unit of Infectious Diseases, Department of Clinical and Experimental Medicine, University of Messina, Messina, Italy; Unit of Infectious and Tropical Diseases, Department of Medical and Surgical Sciences, ‘Magna Graecia’ University of Catanzaro-‘Mater Domini’ Teaching Hospital, Catanzaro, Italy; Department of Medicine and Surgery, Clinic of Infectious Diseases, ‘Santa Maria della Misericordia’ Hospital, University of Perugia, Perugia, Italy; UOC Malattie Infettive, PO Umberto I, Siracusa, Italy; UOC Malattie Infettive, PO Umberto I, Siracusa, Italy; Department of Infectious diseases, AORN Ospedali dei Colli, Cotugno Hospital, Naples, Italy; Department of Infectious diseases, AORN Ospedali dei Colli, Cotugno Hospital, Naples, Italy; Department of Medicine and Surgery, Clinic of Infectious Diseases, ‘Santa Maria della Misericordia’ Hospital, University of Perugia, Perugia, Italy; Department of Health Promotion, Mother and Child Care, Internal Medicine and Medical Specialties, University of Palermo, Palermo, Italy; Unit of Infectious and Tropical Diseases, Department of Medical and Surgical Sciences, ‘Magna Graecia’ University of Catanzaro-‘Mater Domini’ Teaching Hospital, Catanzaro, Italy; Divisione di Malattie Infettive, ASST Grande Ospedale Metropolitano Niguarda, Milan, Italy; Vittorio Emanuele II Hospital, Bisceglie, Italy; Department of Health Promotion, Mother and Child Care, Internal Medicine and Medical Specialties, University of Palermo, Palermo, Italy; University of Bari, Clinic of Infectious Diseases, Bari, Italy; Department of Infectious Diseases, University Hospital ‘Ospedali Riuniti’ of Foggia, Foggia, Italy; Department of Public Health and Infectious Diseases, Sapienza University of Rome, Rome, Italy; Infectious Diseases Unit, Department of Clinical and Experimental Medicine, Azienda Ospedaliera Universitaria Pisana, University of Pisa, Pisa, Italy

## Abstract

**Objectives:**

To describe clinical characteristics and outcomes of COVID-19 patients who developed secondary infections due to carbapenem-resistant Enterobacterales (CRE).

**Methods:**

Retrospective observational study including COVID-19 patients admitted to 12 Italian hospitals from March to December 2020 who developed a superinfection by CRE. Superinfection was defined as the occurrence of documented bacterial infection >48 h from admission. Patients with polymicrobial infections were excluded. Demographic, clinical characteristics and outcome were collected. Isolates were classified as KPC, metallo-β-lactamase (MBL) and OXA-48-producing CRE. A Cox regression analysis was performed to identify factors independently associated with 30 day mortality.

**Results:**

Overall, 123 patients (median age 66 years, IQR 59–75) were included. The majority of infections occurred in the ICU (81, 65.9%), while 42 (34.1%) in medical wards. The most common types of infection were bloodstream infections (BSI) (*n *= 64, 52%), followed by urinary-tract infections (UTI) (*n *= 28, 22.8%), hospital-acquired/ventilator-associated pneumonia (HAP/VAP) (*n *= 28, 22.8%), intra-abdominal infections (*n *= 2, 1.6%) and skin infections (*n *= 1, 0.8%). Sixty-three (51.2%) infections were caused by KPC-, 54 (43.9%) by MBL-, and 6 (4.8%) by OXA-48-producing CRE. Thirty-day mortality was 33.3% (41/123). On Cox regression analysis, HAP/VAP compared with UTI (HR 7.23, 95% CI 2.09–24.97, *P *= 0.004), BSI compared with UTI (HR 3.96, 95% CI, 1.33–11.77, *P *= 0.004), lymphopenia on admission (HR 3, 95% CI 1.44–6.26, *P *= 0.003) and age (HR 1.05, 95% CI 1.02–1.08, *P *= 0.002) were predictors of 30 day mortality.

**Conclusions:**

Superinfections by CRE were associated with high risk of 30 day mortality in patients with COVID-19. HAP/VAP was the strongest predictor of death in these patients.

## Introduction

Since December 2019, COVID-19 has spread across the globe, accounting for more than 5.5 million related deaths, especially in vulnerable patients with comorbidities.^1–3^ The pandemic is placing enormous burdens on global health care and poses many difficulties for appropriate antimicrobial use and stewardship.^4^ As a consequence, bacterial superinfections caused by MDR organisms emerged as an additional challenge during the pandemic and became responsible for mortality and morbidity in hospitalized patients with COVID-19.^5^ Superinfections caused by MDR Enterobacterales, *Pseudomonas aeruginosa* and *Acinetobacter baumannii* have been increasingly reported during the hospital stays of patients with severe COVID-19.^6–8^

Carbapenem-resistant Enterobacterales (CRE) represent a major threat to human health around the world and significantly contribute to global deaths attributable to and associated with bacterial antimicrobial resistance.^9^ Before the COVID-19 pandemic, carbapenem resistance in *Klebsiella pneumoniae* isolates exceeded 50% in some European countries with the highest prevalence of various carbapenemase-producing CRE reported in Israel, Greece, Italy and Turkey.^10^ Although information about the current magnitude of CRE infections in COVID-19 are crucial, only small case series have been reported and limited data are available so far.^11,12^

The aim of this study was to describe clinical characteristics and outcome of COVID-19 patients developing secondary infections due to CRE.

## Patients and methods

### Study design and definitions

This was a retrospective multicentre study including consecutive patients with COVID-19 who developed superinfections caused by CRE admitted to 12 Italian hospitals from 1 March 2020 to 31 December 2020. The research was conducted in accordance with the Declaration of Helsinki and national and institutional standards. The study was approved by the Internal Review Board (IRB) of the Comitato Etico Area Nord-Ovest (CEAVNO, approval number 18214) of the promoter and by local ethics committees of participating centres. Written informed consent was obtained from study participants.

All patients with pneumonia and laboratory-confirmed SARS-CoV-2 infection with a RT–PCR test on a nasopharyngeal swab and with proven superinfection by CRE were eligible. Superinfections were defined as infections that occurred 48 h after admission for COVID-19.^5^ Cases with confirmed infection, defined by the presence of a positive culture of a significant clinical sample, associated with clinical signs of infection and/or worsening organ failure were included.^13^ Conventional microbiological testing (endotracheal aspirate/bronchoalveolar lavage, blood and urine cultures) was requested by the treating physician when infection was suspected. An infection was defined as polymicrobial if another organism was isolated from the same site of CRE infection within 2 days from the CRE culture. Patients with polymicrobial infections were excluded from the final analysis. Superinfections were classified into the following categories: hospital-acquired pneumonia (HAP), ventilator-associated pneumonia (VAP), bloodstream infections (BSI), urinary tract infections (UTI), skin and soft tissue infections, and intra-abdominal infections (IAI), according to CDC/NHSN criteria.^14^ HAP/VAP were defined as pneumonia fulfilling CDC/NHSN Surveillance definitions of healthcare-associated infections for pneumonia with specific laboratory findings.^14^ Epidemiological and demographic information, medical history, comorbidities, information on clinical symptoms at hospital admission and treatments specifically targeting SARS-CoV2 received during the hospital course were collected. Immunomodulant agents included anti-IL-6 (tocilizumab), and Janus kinase inhibitors (JAK) inhibitors (baricitinib). The decision to prescribe one of the two immunosuppressants was taken by the attending physician, according to the national guidelines for the management of patients with COVID-19.^15^ To assess comorbidity burden, the age-adjusted Charlson Comorbidity Index was calculated. Sepsis and septic shock were defined according to the Surviving Sepsis Guidelines and Sepsis-3 consensus.^16^ Severity of infection was assessed using the Pitt bacteraemia score and SOFA score.^17,18^

Day 1 was the day of the positive CRE culture. Ward of hospitalization, oxygen support and presence of septic shock on Day 1 were recorded. Data about antibiotic therapy were also recorded. Appropriate empirical therapy was defined as an antibiotic regimen containing at least one *in vitro* active antibiotic started within 48 h from the culture collection. Targeted antibiotic therapy was categorized into: ceftazidime/avibactam-containing regimens, colistin-containing regimens, other *in vitro* active antibiotics.

All cause in-hospital mortality was recorded. Thirty-day mortality was defined as the occurrence of death within 30 days from Day 1. Data about rectal colonization by CRE at time of CRE infection were collected. Not all participating centres performed a systematic surveillance of CRE-rectal colonization.

MALDI-TOF MS (Bruker Daltonics) was used for species identification. The presence of a *bla* gene, including *bla*_KPC_, *bla*_NDM_, *bla*_VIM_, and *bla*_OXA-48_ was determined by PCR using the GeneXpert System (Cepheid), as previously reported.^19^ Antimicrobial susceptibility tests were performed with the Tecan automated system (Tecan Trading AG, Switzerland) or by Sensititre^TM^ EUMDROXF (Thermo Fisher Scientific Waltham, MA) or Vitek 2 automated system (bioMérieux, Marcy l’Etoile, France) or with the MicroScan system (Beckman Coulter, Brea, CA, USA) according to the manufacturer’s instructions. MICs were classified according to breakpoints established by EUCAST (v10.0).

### Study outcome

The main objective of the study was to describe clinical characteristics and outcomes of patients with COVID-19 who developed superinfections caused by CRE. Accordingly, the primary outcome measure was all-cause 30 day mortality. We also sought to investigate predictors of all-cause 30 day mortality in this patient population.

### Statistical analysis

Continuous variables were presented as the median with IQR. Categorical variables were reported as percentages. A comparison between patients who died and those who survived within 30 days from the superinfection episode was performed. The Mann–Whitney U-test, Chi-square test and Fisher test were used to compare differences between groups, as appropriate. To identify factors independently associated with 30 day mortality, a multivariable Cox regression analysis was performed. The multivariable analysis using Cox regression prediction models was constructed using a forward stepwise procedure, entering all variables with univariate *P *< 0.05 at univariate analysis. Type of infection according to CDC/NHSN definition was included as a categorical variable, using UTI as reference variable. Age was included in the model for its clinical relevance. We screened for collinearity all significant variables identified at the univariate analysis. The variance inflation factor (VIF) value was calculated to control the influence of collinearity. We assumed a lack of multicollinearity if all variables had a VIF value <2. Since collinearity between invasive mechanical ventilation/HAP/VAP was observed, we decided to use the variable ‘type of infection’ in the multivariable model, because of its clinical relevance. Septic shock instead of Pitt bacteraemia score and SOFA score was included in the model to avoid collinearity. All remaining variables displayed no collinearity. The final multivariable model was chosen according to the Akaike information criterion and to parsimony and clinical interpretability of data.

To confirm our findings, a Cox regression analysis using the variable ‘site of infection’ instead of ‘type of infection’ was also built with same above-described methodology.

Finally, sensitivity analyses were performed to evaluate the effect of antibiotic therapy against CRE infections and anti-COVID-19 therapies on 30 day mortality. Statistical significance was established at *P *< 0.05. All reported *P* values are two-tailed. The results obtained were analysed using a commercially available statistical software package (SPSS Statistics version 27.0 software, IBM Corp., Armonk, NY, USA).

## Results

### Study population

**Figure 1.**
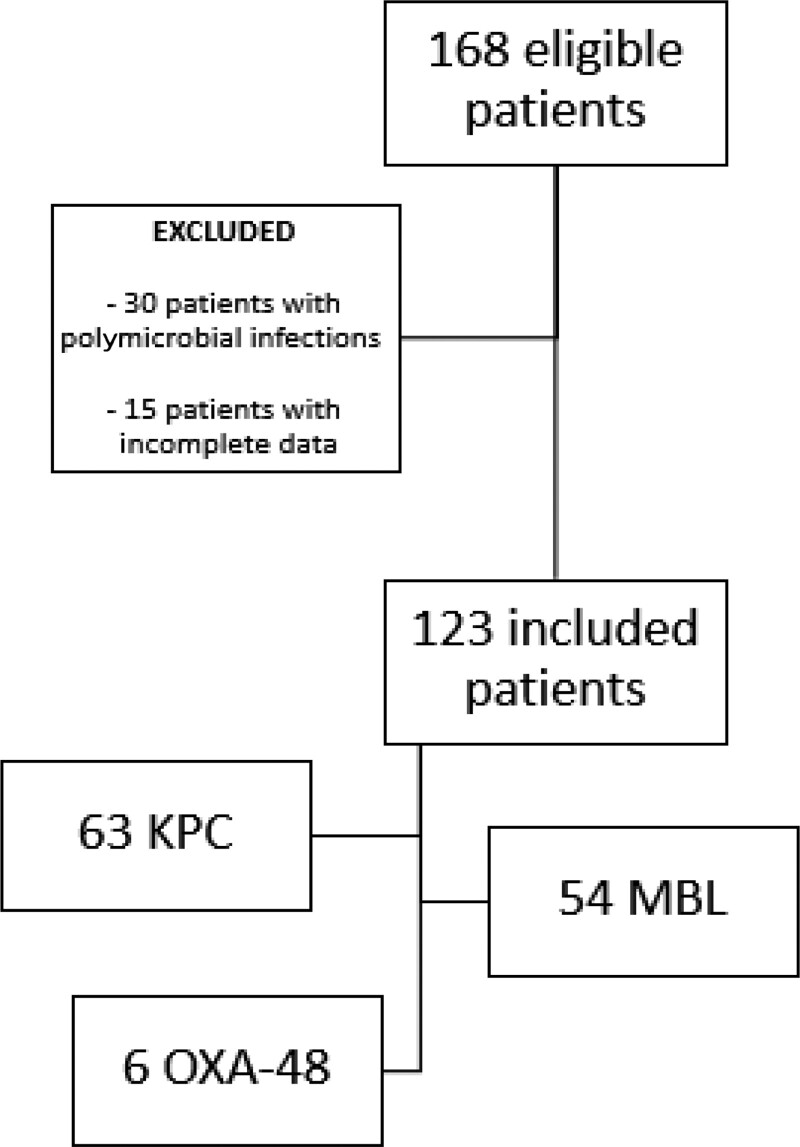
Study flow chart. KPC, *K. pneumoniae* carbapenemase; MBL, metallo-β-lactamase.

A total of 168 patients were eligible for the study: 30 with a polymicrobial infection and 15 with incomplete data were excluded (Figure 1). Hence, 123 COVID-19 patients with CRE superinfections were included. Clinical characteristics and treatments specifically directed against COVID-19 are reported in Table 1. Median age was 66 (IQR 59–75) years and 77.2% of patients were males. The majority were cared for in ICU (81, 65.9%), while the remainder (42, 34.1%) were in medical wards when bacterial superinfection was diagnosed.

**Table 1.** Clinical characteristics and outcome of patients (*N *= 123) with CRE superinfections

Characteristics	Number (%), *N*=123
Demographics	
Age, years, median (IQRs)	66 (59–75)
Male sex	95 (77.2)
Comorbidities	
Cardiovascular disease	48 (39)
Cancer	20 (16.3)
Diabetes mellitus	29 (23.6)
COPD	15 (12.2)
Chronic renal disease	16 (13)
Charlson Comorbidity Score, median (IQR)	3 (1–4)
Clinical features on admission	
PaO_2_/FiO_2_ ratio, median (IQR)	172.5 (126–230)
Thrombocytopenia	38 (30.9)
Lymphocytes < 0.8 × 10^9^/L	63 (51.2)
Treatments against COVID-19	
Corticosteroids	75 (60.9)
Remdesivir	29 (23.6)
Immunomodulants	33 (26.8)
Ward of hospitalization at time of CRE infection	
ICU	81 (65.8)
Medical ward	42 (34.1)
Oxygen support at time of CRE infection	
High flow nasal cannula	3 (2.4)
Non-invasive ventilation	12 (9.8)
Invasive mechanical ventilation	68 (55.3)
CRE rectal colonization^a^	80/106 (75.5)
SOFA, median (IQR) at time of CRE infection	7 (4–11)
Septic shock at time of CRE infection	35 (28.5)
Pitt bacteraemia score at time of CRE infection, median (IQR)	2 (2–4)
Days from admission to superinfection, median (IQR)	16 (10–26)
Time from admission to superinfection, *n* (%)	
3–14 days	47 (38.2)
15–30 days	52 (42.3)
>30 days	24 (19.5)
Length of hospital stay, days, median (IQR)	
From admission	25.5 (16–41)
From infection	15.5 (7–24.75)
All cause in-hospital mortality	51 (41.5)
30 day mortality	41 (33.3)

Abbreviations: ICU, intensive care unit; COPD, chronic obstructive pulmonary disease; CRE, carbapenem-resistant Enterobacterales; SOFA, sequential organ failure assessment.

a Data on rectal colonization was available in 106/123 patients.

**Figure 2.**
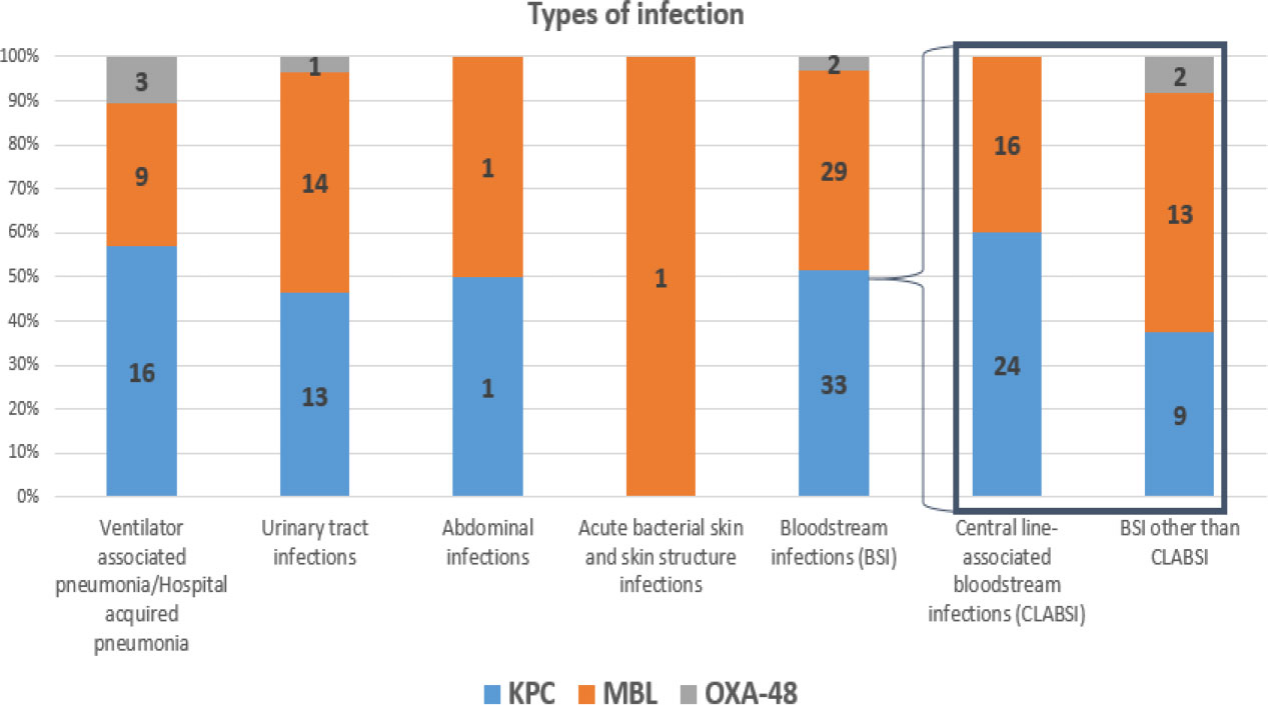
Mechanisms of carbapenem resistance in COVID-19 patients with CRE superinfections according to type of infection. CLABSI, central-line associated bloodstream infections; KPC, *K. pneumoniae* carbapenemase; MBL, metallo-β-lactamase.

The most common types of infection were BSI (*n *= 64, 52%) followed by UTI (*n *= 28, 22.8%), HAP/VAP (*n *= 28, 22.8%), IAI (*n *= 2, 1.6%) and skin structure infections (*n *= 1, 0.8%). Among 64 BSI episodes, the most common source was intravascular catheters (*n *= 40/64, 62.5%), followed by low-respiratory tract (*n *= 13/64, 20.3%), urinary tract (*n *= 10/64, 15.6%) and abdomen (*n *= 1/64, 1.6%). Table 2 reports the aetiology of superinfection episodes. The following species were isolated: *K. pneumoniae* (*n *= 109, 88.6%), *Enterobacter* spp. (*n *= 10, 8.1%), *Proteus mirabilis* (*n *= 3, 2.4%) and *Serratia marcescens* (*n *= 1, 0.8%). Sixty-three (51.2%) infections were caused by KPC-, 54 (43.9%) by MBL- (47 NDM, 7 VIM), and 6 (4.8%) by OXA-48-producing CRE. No isolates producing more than one carbapenemase were identified. Figure 2 reports the aetiology of CRE superinfections according to type of infection.

**Table 2.** Microbial aetiology of episodes of CRE superinfections in hospitalized patients with COVID-19

Characteristic	Number (%), *N *= 123
Species	
* Klebsiella pneumoniae*	109 (88.6)
* Enterobacter* spp.	10 (8.1)
* Proteus mirabilis*	3 (2.4)
* Serratia marcescens*	1 (0.8)
Mechanism of resistance	
KPC-producing CRE	63 (51.2)
MBL-producing CRE	54 (43.9)
OXA-48-producing CRE	6 (4.9)

KPC, *K. pneumoniae* carbapenemase; MBL, metallo-β-lactamase.

Seventy patients (56.9%) received an appropriate empirical antibiotic therapy (Table 3). Patients with infection by KPC-producing CRE were treated with regimens containing ceftazidime/avibactam (50/63, 79.4%), colistin (1/63, 1.6%) or other active antibiotics (11/63, 17.5%). Patients with infection by MBL-producing CRE received a combination of ceftazidime/avibactam plus aztreonam (36/54, 66.7%), colistin-containing regimens (8/54, 14.8%) or other active antibiotics (3/54, 5.6%). All patients with of OXA-48-producing infections received ceftazidime/avibactam. A total of 8/123 (6.5%) patients received no *in vitro* active therapy.

**Table 3.** Treatment of CRE superinfections in hospitalized patients with COVID-19 (*N *= 123)

Characteristic	*n*/*N* (%)
Appropriate empirical therapy	70/123 (56.9)
Targeted antibiotic therapy	
KPC-producing (*n *= 63)	
Ceftazidime/avibactam-containing regimens	50/63 (79.4)
Colistin-containing regimens	1/63 (1.6)
Other active antibiotics	11/63 (17.5)
No active therapy	1/63 (1.6)
MBL-producing (*n *= 54)	
Ceftazidime/avibactam + aztreonam	36/54 (66.7)
Colistin-containing regimens	8/54 (14.8)
Other active antibiotics	3/54 (5.6)
No active therapy	7/54 (13)
OXA-producing (*n *= 6)	
Ceftazidime/avibactam-containing regimens	6/6 (100)

KPC, *K. pneumoniae* carbapenemase; MBL, metallo-β-lactamase.

The 30 polymicrobial infections were caused by: KPC-producing *K. pneumoniae *+ *A. baumannii* (*n *= 16/30, 53.3%), KPC-producing *K. pneumoniae* + *P. aeruginosa* (*n *= 3/30, 10%), KPC-producing *K. pneumoniae* + *Stenotrophomonas maltophilia* (*n *= 2/30, 6.7%), KPC-producing *K. pneumoniae *+ *Enterococcus faecalis* (*n *= 1/30, 3.3%), KPC-producing *K. pneumoniae + Enterobacter cloacae* (*n *= 1/30, 0.33%); MBL-producing *K. pneumoniae *+ *A. baumannii* (*n *= 2/30, 6.7%), MBL-producing *K. pneumoniae *+ *P. aeruginosa* (*n *= 3/30, 10%) and MBL-producing *K. pneumoniae *+ *E. faecalis* (*n *= 1/30, 3.3%). Comparison of patients with monomicrobial and polymicrobial infections is reported in Table S1 (available as Supplementary data at *JAC-AMR* Online). Patients with polymicrobial infections more frequently had HAP/VAP and had higher 30 day mortality rates (56.7% versus 33.3%, *P *= 0.018).

### Outcomes

The 30 day mortality was 33.3% (*n *= 41). Comparison of patients who died within 30 days from superinfection and those who did not is reported in Table 4. On Cox regression multivariable analysis HAP/VAP compared with UTI (HR 7.23, 95% CI 2.09–24.97, *P *= 0.004), BSI compared with UTI (HR 3.96, 95% CI, 1.33–11.77, *P *= 0.004), lymphopenia on admission (HR 3, 95% CI 1.44–6.26, *P *= 0.003) and age (HR 1.05, 95% CI 1.02–1.08, *P *= 0.002) were factors independently associated with 30 day mortality (Table 5). After replacing the variable type of infection with site of infection, the same predictors of 30-day mortality were confirmed (Table S2).

**Table 4.** Comparison of patients who died and those who did not within 30 days from CRE superinfection

Characteristic	Survivors (*N *= 82)	Non-survivors (*N *= 41)	*P* value^a^
Demographics			
Age, years, median (IQR)	65.5 (56–74)	70 (60.5–76.5)	0.131
Male sex, *n* (%)	62 (75.6)	33 (80.5)	0.543
Comorbidities, *n* (%)			
Cardiovascular disease	29 (35.4)	19 (46.3)	0.239
Cancer	9 (11)	11 (26.8)	0.104
Type 2 diabetes	17 (20.7)	12 (29.3)	0.293
COPD	10 (12.2)	5 (12.2)	1
Chronic renal disease	10 (12.2)	6 (14.6)	0.705
Charlson Comorbidity Score, median (IQR)	3 (1–4)	3 (1–4)	0.314
Clinical features on admission			
PaO_2_/FiO_2_ ratio, median (IQR)	180 (123–277)	151 (89–223)	0.075
Thrombocytopenia, *n* (%)	20 (24.4)	18 (43.9)	**0**.**027**
Lymphocytes < 0.8×10^9^/L, *n* (%)	34 (41.5)	29 (70.7)	**0**.**002**
Treatments against COVID-19, *n* (%)			
Corticosteroids	48 (58.5)	27 (65.9)	0.433
Remdesivir	19 (23.2)	10 (24.4)	0.584
Immunomodulant drugs	23 (28)	10 (24.4)	0.985
Ward of hospitalization at time of CRE infection, *n* (%)			**0**.**001**
ICU	46 (56.1)	35 (85.4)	
Medical ward	36 (43.9)	6 (14.6)	
Days from admission to superinfection, median (IQR)	17 (11–28)	15 (7–21)	0.255
Mechanism of resistance, *n* (%)			
KPC	40 (48.8)	23 (56.1)	0.090
MBL	40 (48.8)	14 (34.1)	
OXA-48	2 (2.4)	4 (9.8)	
Oxygen support at time of CRE infection, *n* (%)			
High flow nasal cannula	3 (3.7)	0 (0)	0.550
Non-invasive ventilation	9 (11)	3 (7.3)	0.749
Invasive mechanical ventilation	37 (45.1)	32 (78)	**<0**.**001**
CRE-rectal colonization^b^	53/74 (71.6)	27/32 (84.4)	0.161
Type of infection, *n* (%)			**0**.**005**
UTI	24 (29.3)	4 (9.8)	
BSI	40 (48.8)	24 (58.5)	
VAP/HAP	16 (19.5)	12 (29.3)	
Others	2 (2.4)	1 (2.4)	
Site of infection, *n* (%)			0.050
Urinary tract	29 (35.4)	9 (21.9)	
Catheter-related bacteraemia	30 (36.6)	10 (24.4)	
Respiratory tract	21 (25.6)	20 (48.8)	
Other	2 (2.4)	2 (4.9)	
SOFA, median (IQR) at the time of CRE infection	6 [3–7]	11 [5–13]	**0**.**007**
Septic shock at the time of CRE infection, *n* (%)	17 (20.7)	18 (43.9)	**0**.**007**
Pitt bacteraemia score, median (IQR) at the time of CRE infection	2 (2–2.25)	3 (2–4)	**0**.**003**
Days from admission to superinfection, median (IQR)	17 (12–29)	16 (7–22)	0.330
Days from admission to superinfection, *n* (%)			0.129
3–14 days	28 (34.1)	19 (46.3)	
15–30 days	34 (41.5)	18 (43.9)	
>30 days	20 (24.4)	4 (9.8)	
Appropriate empirical therapy	41 (50)	29 (70.7)	**0**.**029**

ICU, intensive care unit; COPD, chronic obstructive pulmonary disease; CRE, carbapenem-resistant Enterobacterales; SOFA, Sequential Organ Failure Assessment; VAP/HAP, ventilator-acquired pneumonia/hospital-acquired pneumonia; UTI, urinary tract infection.

a Bold indicates statistical significance (*P *< 0.05).

b Data on rectal colonization was available in 106/123 patients.

**Table 5.** Cox multivariable analysis on 30 day mortality

Variable^a^	HR (95% CI)	*P* value
Type of infection		
UTI	Reference variable	–
BSI	3.96 (1.33–11.77)	0.013
HAP/VAP	7.23 (2.09–24.97)	0.002
Others	1.16 (0.13–10.68)	0.899
Lymphopenia on admission	3 (1.44–6.26)	0.003
Age	1.05 (1.02–1.08)	0.002

HAP/VAP, hospital-acquired pneumonia/ventilator-associated pneumonia; BSI, bloodstream infection.

a Variables included in the model and not retained: thrombocytopenia (defined as platelets <150×10^9^/L) on admission; source control; septic shock.

A first sensitivity analysis taking into account antibiotic therapy confirmed that age (HR 1.05, 95% CI 1.02–1.09, *P *< 0.001), HAP/VAP compared with UTI (HR 9.45, 95% CI 2.57–34.71, *P *< 0.001) and BSI compared with UTI (HR 5.73, 95% CI 1.78–18.38, *P *= 0.003) were factors independently associated with 30 day mortality (Table S3).

A second sensitivity analysis taking into account anti-COVID-19 therapies confirmed that age (HR 1.05, 95% CI 1.01–1.08, *P *= 0.008), HAP/VAP compared with UTI (HR 5.23, 95% CI 1.4–19.57, *P *= 0.014) and BSI compared with UTI (HR 3.48, 95% CI 1.02–11.89, *P *= 0.046), lymphopenia (HR 2.45, 95% CI 1.11–5.43, *P *= 0.026) and thrombocytopenia on admission (HR 2.4, 95% CI 1.17–4.93, *P *= 0.017) were factors independently associated with 30 day mortality (Table S4).

## Discussion

Our observational study describes the clinical characteristics and outcome of patients with COVID-19 with superinfections caused by CRE in a multicentre national cohort. We found that patients who developed a superinfection caused by CRE had a substantial risk of mortality after the infection episode. Moreover, respiratory tract infection (HAP/VAP) was the strongest predictor of poor outcome in these patients.

Mortality rate within 30 days from CRE superinfection was as high as 33.3%. In a previous study conducted during the first wave of the pandemic we found a mortality rate of 23% subsequent to a superinfection caused by any MDR organism,^5^ while we recently reported a mortality rate >45% in patients with COVID-19 who developed superinfections caused by ESBL-producing hypervirulent *K. pneumoniae*.^7^ Debates continue to exist about cause of mortality and proportion of deaths formally attributable to COVID-19. It should be underlined that, beyond deaths due to respiratory failure because of COVID-19, superinfections caused by MDR organisms may complicate the course of hospital stay and substantially increase the risk of death in COVID-19 patients. As a matter of fact, a recent review of post-mortem studies showed that histopathologic findings were consistent with lung superinfections in 32% of COVID-19 patients.^20^ Unfortunately, limited data about the proportion of MDR organisms causing superinfections are available in autopsy studies.^20^ The impact of superinfections caused by MDR organisms on the outcome of hospitalized patients with COVID-19 should be further investigated.

In a prospective cohort study of critically ill, ventilated COVID-19 patients, the presence of a bacterial pulmonary superinfection was associated with extended ventilation times, increased duration of ICU hospitalization, and increased need for intensive-care rescue therapy.^21^ Our study confirmed that HAP/VAP caused by CRE is a factor independently associated with mortality, even after adjustment for relevant confounders such as patient-related (age), treatment-related factors (source control, appropriate empirical therapy and type of targeted therapy) and severity of COVID-19. Furthermore, we also found that patients with polymicrobial infections more frequently had HAP/VAP and had higher 30 day mortality compared with monomicrobial infections. In the subgroup of patients with HAP/VAP caused by CRE, 39.1% had polymicrobial infections with CRE isolated together with non-fermenting Gram-negative or other bacteria. In line with our results, a recent multicentre retrospective study showed that polymicrobial VAP accounted for 39.0% of all VAP in ICU, and Enterobacterales accounted for 49.8% of all the isolated pathogens.^22^ Discrimination between pathogens and colonizer in these cases is not easy. It is well recognized that diagnosis of VAP in severe COVID-19 may be challenging, since no standardized diagnostic exists and there could be a risk of VAP overdiagnosis in critically ill COVID-19 patients.^23,24^ The isolation of multiple bacteria from the respiratory tract of these patients may lead to antibiotic overuse and hence paradoxically increase the selective pressure for MDR organisms. However, given the significant related mortality, the early recognition of patients with HAP/VAP is crucial. Microbiological surveillance with periodic endotracheal aspirates in intubated patients may be useful for the early detection of CRE and for a better understanding of the colonization status of the patient. However, clinicians should carefully correlate microbiological findings with laboratory/clinical parameters to avoid unnecessary antibiotic pressure in patients with respiratory colonization.

Importantly, we noted that the majority of BSI were associated with intravascular catheters. Moreover, in our study superinfections by CRE occurred after at least 2 weeks from hospital admission. Not surprisingly, prolonged hospitalization favours the development of infections caused by MDR organisms. One-third of patients with CRE superinfections were being cared for in medical wards when the infection episode occurred. All together, these findings highlight the difficulties for healthcare workers in adhering to standard infection prevention and control (IPC) precautions.^25^ The spread of CRE also outside the ICU during the COVID-19 pandemic is concerning and highlights the need for infection control measures both in the ICU and in medical wards.

At the beginning of the pandemic, personal protective equipment (medical gowns, latex gloves, and surgical masks) were employed by health care personnel as well as the disinfection of surfaces and frequent hand washing. However, the focus of healthcare workers on self-protection rather than on preventing cross-transmission from patient to patient has been identified as one of the causes of rapid spread of MDR organisms in COVID-19 wards.^25^ Moreover, traditional IPC efforts, such as screening for carriage of MDR organisms and cohorting of colonized patients, may have been temporarily discontinued during the pandemic.^25^ In addition, other factors, including hospital overcrowding, the misuse of antibiotics and the use of steroids and immunomodulant drugs, all contributed to the increasing risk of MDR spread in the hospital in the era of COVID-19.^25^ The CREVID study highlights the urgency of implementing both infection control measures and antimicrobial stewardship practices in COVID-19 wards, especially in Italy, a country with high prevalence of resistant organisms. Adherence to all IPC measures should be strengthened, including hand washing, changing gloves from patient to patient, active surveillance, and cohorting of colonized patients. A national action plan to implement surveillance and educational programmes, to facilitate the use of rapid and innovative diagnostic tests for identification and characterization of resistant bacteria, and to improve international collaboration on AMR prevention and control is crucial to contain the spread of MDR organisms. Monitoring the epidemiology of specific types of CRE (MBL and non-MBL-producing Enterobacterales) at local and national level also represents a urgent priority.

One of the participating centres, the University Hospital of Pisa, adopts a periodic and systematic screening of rectal colonization for CRE in hospitalized patients together with a microbiology service available 24 h daily 7 days a week (microbiology 24/7).^19^ This strategy leads to the best knowledge of colonization status in hospitalized patients, reduces the time to appropriate antibiotic treatment in septic patients and allows an early de-escalation in case of susceptible pathogens being isolated.^26^ Although cost-effectiveness should be further evaluated, this model may be useful for the optimal management of hospitalized patients in settings with a high prevalence of CRE, especially in the post-COVID-19 era. Finally, promoting outpatient management of COVID-19 would be ideal to reduce the transmission of MDR organisms in hospitals and the impact of antimicrobial resistance on healthcare systems that are already in crisis under the cumulative toll of the spread of the omicron variant of concern. Thus, outpatient management of COVID-19 should be implemented to avoid hospitalization and all related complications, including the risk of bacterial superinfections.^27^

Our study has several limitations. First, the observational and retrospective nature imply a risk of selection bias. We tried to decrease this effect with a fast review of included patients by the participating centres in order to identify possible issues regarding relevant and mandatory data. Second, heterogeneity in the diagnosis and management of CRE superinfections may have occurred between the different centres. Third, although Italy is a country with a high prevalence of CRE, we were only able to include a relatively low number of patients with CRE infections. This may be due to a change in the epidemiology with an increased number of non-fermenting Gram-negative bacteria (GNB) during COVID-19 in Italian hospitals. However, we cannot provide data about the prevalence of CRE infections. Thus, this study should not be used to estimate the actual prevalence of CRE infections. Further studies should provide more detailed epidemiological data about the spread of both CRE and non-fermenting GNB during COVID-19. Finally, in our cohort only 61% of patients received steroids. This may be because this study was conducted from March to December 2020, whereas data from the RECOVERY Study showing the efficacy of dexamethasone in hospitalized patients with COVID-19 were released in June 2020 and regulatory agencies of drugs authorized the use of steroids in September/October 2020. In any event, a sensitivity analysis taking into account the anti-COVID-19 treatments confirmed our study results.

In conclusion, we described the characteristics and outcome of patients with COVID-19 who developed a superinfection by CRE, highlighting that the spread of CRE occurring both in the ICU and in medical wards represents a relevant challenge for clinicians. Patients with CRE superinfections displayed a significant risk of mortality after the infection episode and lower respiratory tract infection is one of the main predictors of poor outcome in these patients. Thus, systematic surveillance of CRE colonization and early diagnosis of CRE superinfections should be implemented in patients with COVID-19 both in the ICU and in medical wards.

## Supplementary Material

dlac064_Supplementary_Data

## Transparency declarations

M.F. received grants and/or speaker honoraria from MSD, Angelini, Shionogi, Pfizer, Menarini, Gilead and Nordic Pharma. F.M. has participated in advisory boards and/or received speaker honoraria from Angelini, Correvio, Merck Sharp & Dohme (MSD), Nordic Pharma, Pfizer, Astellas, Gilead, Bristol-Myers Squibb (BMS), Janssen, ViiV, bioMérieux, Biotest, Becton Dickinson, Pfizer, and Shionogi. The declared conflicts of interest are outside the submitted work and did not affect the scientific objectivity of this study. The other authors have none to declare.

## Supplementary data

Tables S1 to S4 are available as Supplementary data at *JAC-AMR* Online.
