# Supplementary material for: Superinfections caused by carbapenem-resistant Enterobacterales in hospitalized patients with COVID-19: a multicentre observational study from Italy (CREVID Study)
Source: JAC Antimicrob Resist. 2022 Jun 16;4(3):dlac064. doi: 10.1093/jacamr/dlac064 (PMC9201238; doi:10.1093/jacamr/dlac064)
Supplement: dlac064_Supplementary_Data [file dlac064_supplementary_data.docx]

**Supplementary data**

**TABLE S1.** Comparison mono- and polymicrobial infections main characteristics

|  | **Total**  **N =153 (%)** | **Monomicrobial**  **N= 123 (%)** | **Polymicrobial**  **N= 30 (%)** | **p-value** |
| --- | --- | --- | --- | --- |
| **DEMOGRAPHICS**  Age, median (IQRs)  Male sex | 66 (57.5-75.5)  114 (74.5) | 66 (59-75)  95 (77.2) | 63 (55.7-77.2)  19 (63.3) | 0.758  0.117 |
| **COMORBIDITIES**  Cardiovascular disease  Cancer  Diabetes mellitus  COPD  Chronic Renal disease  Charlson Comorbidity Score, median (IQRs) | 63 (41.2)  22 (14.4)  33 (21.7)  18 (11.8)  21 (13.7)  3 (2-5) | 48 (39)  18 (14.6)  29 (23.6)  15 (12.2)  16 (13)  3 (2-5) | 15 (50)  4 (13.3)  4 (13.3)  3 (10)  5 (16.7)  3 (1-5) | 0.280  0.856  0.250  1  0.602  0.823 |
| **CLINICAL FEATURES ON ADMISSION**  PaO_2_/FiO_2_ ratio, median (IQR)^a^  Thrombocytopenia^b^  Lymphocytes < 0.8 x 10^9^/l^b^ | 168 (104-252)  45 (30)  84 (56) | 172 (115-265)  38 (31.1)  63 (51.6) | 117 (80-211)  7 (25)  21 (75) | 0.131  0.522  ***0.025*** |
| **TREATMENTS AGAINST COVID-19**  Corticosteroids  Remdesivir  Immunomodulant drugs | 90 (62.5)  31 (22.5)  39 (27.7) | 75 (61)  29 (25.2)  33 (28.4) | 15 (71.4)  2 (8.7)  6 (24) | 0.360  0.104  0.652 |
| **WARD OF HOSPITALIZATION AT TIME OF CRE INFECTION**  ICU  Medical ward | 98 (64.1)  55 (35.9) | 81 (65.9)  42 (34.1) | 17 (56.7)  13 (43.3) | 0.347 |
| **OXYGEN SUPPORT AT TIME OF CRE INFECTION**  High Flow Nasal Cannula  Non Invasive Ventilation  Invasive mechanical ventilation | 24 (15.7)  12 (7.8)  78 (51) | 3 (2.4)  12 (9.8)  69 (56.1) | 21 (70)  0 (0)  9 (30) | ***<0.001***  *-*  ***0.01*** |
| **TYPE OF INFECTION**  UTI  BSI  VAP/HAP  Others | 32 (21)  72 (47)  46 (30)  3 (2) | 28 (22.8)  64 (52)  28 (22.8)  3 (2.4) | 4 (13.3)  8 (26.7)  18 (60)  - | ***0.030*** |
| CRE-rectal colonization | 92 (60) | 80 (65) | 12 (40) | ***0.013*** |
| Central venous catheter | 137 (89.5) | 110 (89.4) | 27 (90) | 1 |
| Septic shock | 44 (28.7) | 35 (28.5) | 9 (30) | 0.993 |
| All cause in-hospital mortality  Thirty-day mortality | 71 (46)  58 (38) | 51 (41.5)  41 (33.3) | 20 (66.7)  17 (56.7) | ***0.015***  ***0.018*** |

^a^ Data available for 132 patients

^b^ Data available for 150 patients

**TABLE S2.** Cox multivariable analysis on 30-day mortality (using site of infection instead of type of infection classified according CDC/NHSH definition).

| **Variable** | **HR (95% CI)** | ***p-value*** |
| --- | --- | --- |
| Site of infection  Urinary tract  Catheter-related bacteremia  Respiratory tract  Others | Reference variable  1.96 (0.76-5.04)  3.61 (1.39-9.38)  0.8 (0.16-3.95) | -  0.16  0.008  0.789 |
| Lymphopenia on admission | 2.86 (1.31-6.23) | 0.008 |
| Age | 1.05 (1.02-1.08) | 0.003 |

Variables included in the model and not retained: thrombocytopenia (defined as platelets <150 x 10^9^/l) on admission; source control, septic shock.

Legend: HAP/VAP: Hospital acquired pneumonia/Ventilator associated pneumonia; BSI: bloodstream infection

**TABLE S3.** Cox multivariable analysis on 30-day mortality (entering antibiotic therapy in the model).

| **Variable** | **HR (95% CI)** | ***p-value*** |
| --- | --- | --- |
| Site of infection  UTI  BSI  HAP/VAP  Others | Reference variable  5.73 (1.78-18.38)  9.45 (2.57-34.71)  1.67 (0.16-17.36) | -  0.003  <0.001  0.665 |
| Lymphopenia on admission | 2.16 (0.82-5.96) | 0.119 |
| Age | 1.05 (1.02-1.09) | <0.001 |
| Appropriate empirical therapy | 1.53 (0.59-3.9) | 0.374 |
| Targeted antibiotic therapy  No active antibiotic therapy  Colistin-containing regimens  CZA-containing regimens  Other regimens | Reference variable  0.001 (0.9-1.01)  0.59 (0.18-1.98)  0.74 (0.17-3.28) | -  0.977  0.394  0.695 |

Variables included in the model and not retained: thrombocytopenia (defined as platelets <150 x 10^9^/l) on admission; source control, septic shock

**TABLE S4.** Cox multivariable analysis on 30-day mortality (entering anti-COVID-19 therapy in the model).

| **Variable** | **HR (95% CI)** | ***p-value*** |
| --- | --- | --- |
| Site of infection  UTI  BSI  HAP/VAP  Others | Reference variable  3.48 (1.022-11.89)  5.23 (1.4-19.57)  0.82 (0.07-8.88 | -  0.046  0.014  0.869 |
| Lymphopenia on admission | 2.45 (1.11-5.43) | 0.026 |
| Age | 1.05 (1.01-1.08) | 0.008 |
| Thrombocytopenia on admission | 2.4 (1.17-4.93) | 0.017 |
| Source control | 0.56 (0.24-1.31) | 0.180 |
| Septic shock | 1.69 (0.74-3.85) | 0.211 |
| Dexamethasone | 1.59 (0.69-3.68) | 0.269 |
| Immunomodulators | 0.564 (0.23-1.39) | 0.212 |
